# The Danger of Long‐Term Use of *Rauwolfia vomitoria* Afzel. (Apocynaceae) Aqueous Root Back Extract for Benign Prostatic Hyperplasia

**DOI:** 10.1155/bmri/2449997

**Published:** 2025-09-18

**Authors:** Perpetua Dagadu, Samuel Adjei, George Awuku Asare, Kwasi Bugyei, Rodger Apandago Mahamadu, Ufuoma Ohwo, Habibur Rahman

**Affiliations:** ^1^ Department of Medical Pharmacology, University of Ghana Medical School, Accra, Ghana, ug.edu.gh; ^2^ Department of Animal Experimentation, Noguchi Memorial Institute for Medical Research (NMIMR), University of Ghana, Accra, Ghana, ug.edu.gh; ^3^ Department of Medical Laboratory Sciences, University of Ghana, Accra, Ghana, ug.edu.gh; ^4^ Department of Medical Laboratory Science, Novena University Ogume, Ogume, Delta State, Nigeria; ^5^ Department of Pathology, Bangladesh Agricultural University, Mymensingh, Bangladesh, bau.edu.bd

**Keywords:** hepatotoxicity, plant extract, *Rauwolfia vomitoria*, safety, sub-chronic toxicity

## Abstract

*Rauwolfia vomitoria* has recently been reported as a promising phytomedicine for benign prostatic hyperplasia (BPH). It has a wide range of therapeutic advantages intermingled with diverse controversies of toxicities, necessitating the need to proceed on a long‐term investigation to determine the safety of *R. vomitoria*. The study is aimed at determining the subchronic toxicity of *R. vomitoria.* Rats were randomised into four (4) groups, which included the normal control group (C), *R. vomitoria* root bark aqueous extract (RVRAE), low dose (LD, 10 mg/kg bwt.), medium dose (MD, 25 mg/kg bwt.) and high dose (HD, 50 mg/kg bwt.). The experimental set‐up included daily administration of plant extracts for a period of 90 days. Relative organ weights, haematological and renal function revealed no significant differences across the treatment groups. However, for liver function, whilst most liver analytes remained unchanged, a significant increase in alkaline phosphatase (ALP) was observed across treatment groups. *C* and LD values were *C* = 144.2 ± 29.3 and LD = 246.4 ± 66.9 (IU) (*p* = 0.008). Total bile acids (TBAs) reduced in a dose‐dependent manner; *C* = 27.9 ± 7.6, LD = 19.0 ± 5.5, MD = 18.6 ± 4.3, HD = 116.8 ± 16.8 * μ*mol/L. The most prominent significant value among others occurred between the C and HD groups (*p* = 0.004). Absolute and relative organ weights of lungs decreased in a dose‐dependent manner. However, only the absolute organ weight was significant (*p* < 0.05) with values of *C* = 2.13 ± 0.12, LD = 1.81 ± 0.05, MD = 1.77 ± 0.15, HD = 0.62 ± 0.17 g. PSA levels in the study did not show significant differences (*p* > 0.05). However, a decline was observed with the high dose group. No significant histopathological alterations were observed in the kidneys, confirming the absence of renal toxicity. However, some histoarchitecture alterations were observed in the liver and lungs, which require further investigation. The safety of the root bark extract remains doubtful, with the lungs and the liver adversely affected even at lower doses of 10 mg/kg bwt.

## 1. Introduction

*Rauwolfia vomitoria* has recently been reported as a promising phytomedicine for benign prostatic hyperplasia (BPH) [1, 2], despite the total absence from literature of any anecdotal evidence from any racial group. It grows extensively across Africa, from Cameroon to Senegal as well as Egypt. Further across East Africa, it grows in Sudan, Uganda and Zaire. This plant will usually not grow beyond 10 m as a shrub. Its fruits are small and reddish in colour and of the size of 5–8 mm, with small seeds of about 3 mm in diameter. The flowers of *R. vomitoria* are usually small and cream in colour with an aromatic smell bearing petals not longer than 10 cm. Its purposes are numerous. Nonmedical purposes include fencing and serving as avenue trees in Kenya and shade‐bearing trees for young cacao trees in Gabon. Young twigs are used for drinks [2]. In the Ivory Coast, the Serpent `Sect of the Man Region recognises the plant for its fetish purposes [3].

Medicinal purposes include the roots being used as a decoction to aid neural relaxation and sleep [4]. Root back decoctions are given as a sedative in small doses to children in the Ivory Coast [5]. Herbal medicine practitioners in Ghana and Nigeria use the root back extract for treating jaundice [6], whilst in the Ivory Coast, *R. vomitoria* is used for UTI treatment. In Congo, the powdered back is used for skin diseases [7]. *R. vomitoria* extract is said to contain *Rauwolfia serpentina* that has hypotensive effects. Reserpine, also found in the root back, is an alkaloid that has antihypertensive properties [8].

More studies in recent times have reported the hypoglycaemic and antihyperglycaemic effects, justifying the use of *R. vomitoria* in the traditional management of diabetes. The aqueous extract was administered in doses of 500, 700 and 1000 mg/kg bwt alongside 10 mg/kg glibenclamide to high glucose‐fed diabetes mellitus (DM)‐induced rats (4000 mg/kg bwt). The glucose tolerance in the rats was rapidly diminished after *R. vomitoria* administration [9].

The cholesterol‐lowering effect of the methanolic leave extract of *R. vomitoria* was demonstrated when rats were fed a high cholesterol diet for 45 days to induce hyperlipidaemia alongside *R. vomitoria* (100–200 mg). Malondialdehyde (MDA) was elevated, and the high phenolic antioxidant activity in the leaf extract reduced tissue MDA and reversed plasma lipids to near normal levels [10].

The aqueous leaf extract of *R. vomitoria* has been purported to have aphrodisiac effects as demonstrated with Wistar rats. The ride, coitus and ejaculatory frequency and latency were all reported to be significant (*p* < 0.001) after 8 days of continuous treatment with 500 and 1000 mg/kg bwt of the extract. It was further alluded that the aphrodisiac potential is higher at higher doses of 1000 mg/kg bwt [11]. Furthermore, toxicity was not observed at a single dose of 2000 mg/kg bwt.

Improvement in testosterone levels was observed with an aqueous ethanolic extract that was administered to rats at 20, 40 and 100 mg/kg bwt for 60 days. At a dose of 40 mg/kg bwt, the weight of reproductive organs increased. Furthermore, serum testosterone increased significantly. Testicular antioxidants such as thiobarbituric acid (TBA), glutathione and catalase also increased. Thus, the study suggests male fertility improvement with *R. vomitoria* [12].

Other health benefits of *R. vomitoria* have been reported to include immunity improvement. This was observed when 150 and 300 mg/kg bwt root bark extracts were administered to rats and haematological indices examined. A decline in WBC was observed [13].

The antitumour effect on cell lines and ovarian cancer models was evaluated using 20 and 50 mg/kg bwt on mice. The growth of tumour was suppressed by 36% and 66%, respectively, in the mice. Furthermore, there was synergistic interaction potentiating the effect of carboplatin [14].

It has been reported that 16 mg/kg bwt *R*. *vomitoria* root back aqueous extract treatment for 15 days is capable of restoring impaired learning and memory as demonstrated by 3‐nitropropionic acid‐induced oxidative stress in mouse models [15]. However, closely related is the report on the anticonvulsant activity. At 200 mg/kg, the aqueous leaf extract prolonged the onset of convulsion in male albino mice [15].

A ß‐carboline alkaloid alstonine, found in the root bark extract, had reserpine removed. Deionised water was used to dissolve the *R. vomitoria* powder and subsequently tested on in vivo and in vitro systems. *R. vomitoria* was able to decrease LNCaP prostate cancer cell growth significantly in a dose‐dependent manner [16]. In in vivo studies using grafted tumour cells in male nude mice, the study demonstrated shrinkage in tumour volumes ranging from 58% to 70% with the lowest and highest dose, 7.5–75 mg/kg bwt *R. vomitoria* administered, but not in a dose‐dependent manner [16]. When BPH animal models were produced in rats, *R. vomitoria* reduced 5‐alpha reductase and consequently reduced prostate volume by increasing the prostate lumen upon reduction of the epithelial lining, as well as the androgen receptors. These effects were very similar to finasteride, which was used as a positive control. Despite these changes, sperm count was not affected [1].

Genotoxic and cytotoxic assessments using male germ cell lines of the pest grasshopper *Zonocerus variegatus* (Orthoptera: Pyrgomorphidae) with the aqueous stem back extract were performed. Positive results obtained demonstrated significant genotoxic and cytotoxic activities [17].

Toxicity studies especially on the liver and kidney have been examined in different studies. Ethanolic extracts of the leaf and root were orally administered to rats daily at 1 and 2 g/kg bwt for 14 days. Liver and kidney function tests were performed, and relative organ weights were determined. Both blood and tissue samples did not reveal hepatorenal toxicity [18].

Chronic toxicity studies on Wistar rats for 60 days using the aqueous root extract of *R. vomitoria* Afzel. (Apocynaceae) were performed. This was followed by the cessation of drug administration for a month. *R. vomitoria* at 700 mg/kg bwt did not affect haematological parameters. However, at doses of 1000 mg/kg bwt, erythrocytes, platelets and leukocytes were affected. The effects were reversible after cessation of treatment [19].

Similar studies were undertaken to assess the safety of *R. vomitoria* ethanolic leaf and root extract. Extracts were administered by gastric intubation for 7 days. Using a dose of 524 mg/kg bwt, the root extract showed greater toxicity than the leaf extract, by AST and ALT increases [20]. Further in vivo work using cervical ventral horn cells had adverse effects when treated with 200, 300 and 400 mg/kg bwt 80% ethanol root back extracts. Hypertrophy of the central horn neurons was observed. Further to this, some ventral horn neurons demonstrated hyperplasia and karyorrhectic appearance [21].

The crude ethanolic extract on foetal lungs at 150 and 250 mg/kg bwt leaf extract showed marked distortion of the architecture of the lungs in Wistar rats [22].

When aqueous stem back of *R. vomitoria* was administered to Wistar rats at doses of 300, 600 and 900 mg/kg bwt for 28 days, liver markers such as AST and ALT were significantly elevated. Furthermore, WBC increased by 52%. However, the histology of the liver was found to be normal, and the authors concluded that the aqueous extract of *R. vomitoria* had no adverse effect on the liver and kidney [23].

The active pharmacological agents determined by GC‐MS showed 22 and 16 phytochemicals in the leaf and roots, respectively. Squalene > phytol > n − hexadecanoic acid > tetradecenal > 9, 12, 15 − octadecatrienoic acid > ethyl ester. The roots contained cis − vaccenic acid > n − hexadecanoic acid > ( E) − 9 − octadecenoic acid ethyl ester > cyclohexanecarbonitrile > 1 − (−4 − chlorophenyl) > 8H − azeceno [5, 4 − b] indol − 8 − one > 5 − ethylidene. Thus, the therapeutic effect may be greater in the leaf than in the roots [24].

The compelling therapeutic advantages intermingled with diverse controversies of toxicities, necessitated the need to proceed on a long‐term investigation to determine the safety of *R. vomitoria*.

## 2. Material and Methods

### 2.1. Plant Material

#### 2.1.1. Plant Collection and Identification

Plant roots of *R. vomitoria* were harvested from Swedru in the central region of Ghana (5.38 N; 0.45 W off Nsabaa Road) on 13th October 2023 (between 4 and 5 pm). The plants were identified by their vernacular name *Kakapenpen* by the farmers, after preliminary identification using a Google plant app. Further authentication by a taxonomist, Mr. Francis Okine Asamoah of the University of Ghana herbarium, was undertaken, and Voucher No. 11062b was issued.

#### 2.1.2. Preparation of Aqueous Extract of *R. vomitoria*

##### 2.1.2.1. Plant Extracts

The roots were carefully washed with water, subdued‐sun‐dried for 2 weeks, pulverised, packaged in sample bottles, labelled appropriately, and stored at room temperature (25°C–27°C) prior to the onset of the experiment. The powdered rootbark of *R. vomitoria* was weighed to obtain one thousand grams (1000 g). The selected drying method was based on other studies. Asoro et al. [25] concluded that sun‐drying, oven‐drying and freeze‐drying did not affect bioflavones and bioactive compounds in ginkgo leaves. Others have argued that with regard to antioxidant activity and flavonoid preservation, sun‐drying and freeze‐drying are the best methods for mulberry leaves, for example [26]. However, freeze‐drying was recommended for leaves of certain plants such as green tea, *Carica papaya*, and guava, in order to attain the desired amounts of flavonoids and polyphenols [27–30]. Sun‐drying is often slower and longer, and perhaps more natural to the plant’s environmental conditions of humidity and temperature. Additionally, it is more cost‐effective than oven‐drying. Other researchers such as Ekong et al., using *R. vomitoria* roots (as used in this study), resorted to air‐drying [31]. Similarly, air‐dried clean root bark of *R. vomitoria* has been employed by others, Ye et al. [32]. However, Eluwa et al. dried *R. vomitoria* in a carbolite moisture extraction drying oven at 40°C–50°C for 3 h [33]. Indeed, drying methods differ for many natural products including *R. vomitoria*. Agreeably, it will be worthwhile exploring the best drying technique followed by phytochemical analysis to determine what is best for future research of *R. vomitoria*.

The rootbark was macerated separately for 24 h with 4000 mL of distilled water and was heated for 1 h afterwards [34]. The extract obtained was filtered through a sterile gauze to separate it from the residue. Another 3000 mL of distilled water was added to the residue and macerated for a further 24 h [34]. The above procedure was repeated to obtain a second extract. The extracts were then pooled and freeze‐dried using Freeze Dryer Gamma 1‐16/2‐16‐LSC 2004. Subsequently, the dried yield was weighed and stored in a sealed container in a refrigerator between 2°C and 8°C until it was used [34]. In this study, the rootbark was macerated separately for 24 h. Although a higher extraction efficiency at 48‐h maceration period may be useful, other risk factors may accompany the longer extraction duration. If the extraction time is longer, undesirable constituents may also be extracted [35]. For example, waxes and tannins that may not be desirable for this study will accompany a longer extraction time [36]. Although a longer extraction time may result in higher yields, a longer extraction time can also lead to degradation of some compounds and the possible formation of unwanted compounds [37] which could possibly affect taste, colour and stability of the extract. Additionally, the protocol [34] which was used has been effective over the years. One could explore a longer extraction time in the future.

### 2.2. Experimental Animals

Rats were obtained and housed at the Department of Animal Experimentation (DAE), Noguchi Memorial Institute for Medical Research (NMIMR), University of Ghana. Animals were allowed to acclimatise for 7 days in plastic cages with wire screen tops at room temperature. Wood shavings were used as bedding in accordance with ethical guidelines. Rats were made to be under 12‐h light and 12‐h darkness. Temperature and humidity were set at 22^°^C ± 3^°^C and 40%–45%, respectively. Furthermore, rats were fed commercial rat chow and watered *ad libitum* throughout the study [38].

#### 2.2.1. Experimental Design

Experimental procedures were carried out in accordance with international ethics guidelines on animal care, the National Institutes of Health Guide for the care and use of laboratory animals and the University of Ghana‐Institutional Animal Care and Use Committee’s (IACUC) guidelines. The study was an experimental design that included the use of 54 adult pathogen‐free male Sprague‐Dawley (SD) rats weighing 300–400 g.

### 2.3. Experimentation

Rats were randomised into four (4) groups which included the normal control group (group C). A stratified randomisation method was used. Basically, all rats were labelled, weighed and ranked from the lowest to the highest. They were divided into weight strata of quantiles. From each weight stratum, one rat was selected for a group, thus ensuring a fair distribution of weights across groups. By so doing, biases were minimised. The experimental setup included daily administration of plant extract *R. vomitoria* root bark aqueous extract (RVRAE), low dose (LD, 10 mg/kg bwt), RVRAE medium dose (MD, 25 mg/kg bwt), and *RVRAE* high dose (HD, 50 mg/kg bwt) for a period of 90 days. The C group was given distilled water, and all animals fed the chow diet ad libitum.

### 2.4. Termination of Experiment

Rats were anaesthetised using isoflurane gas administered within a chamber. The chamber was rapidly saturated with 4%–5% isoflurane (Pharmanova, India) gas. A 10‐L chamber was used with a flow rate of about 5 L per minute. The chamber was carefully controlled to ensure the appropriate concentration of isoflurane was attained. An amount of 5 mL of blood was drawn by cardiac puncture and discharged into EDTA and heparinised Eppendorf tubes. Rats were then euthanised using isoflurane in a glass euthanasia chamber and observed for death as an end point. Organs harvested for evaluation included prostate, seminal and other systemic organs such as the liver, kidneys, heart, lungs, spleen and pancreas. Organs were weighed, rinsed in normal saline solution and placed in 10% buffered formalin for histological analysis.

#### 2.4.1. Laboratory Test

##### 2.4.1.1. PSA (Principle and Methods)

PSA analyses were carried out using the MyBiosource Rat PSA ELISA kit (San Diego, USA). This ELISA kit employed the Sandwich‐ELISA principle. The manufacturer’s instructions were followed. The microwells of the ELISA plate were precoated with an antibody specific to rat PSA. Samples and standards were added to the ELISA plate wells, which combined with the PSA in the serum. A biotinylated detection antibody specific for rat PSA and avidin‐horseradish peroxidase (HRP) conjugate were added successively to each microplate well and incubated. Unbound components were washed away. Substrate solution was added and incubated. The enzyme–substrate reaction that occurred subsequently was terminated by the addition of a stop solution (Conc. HCl). The optical density (OD) was measured spectrophotometrically at a wavelength of 450 ± 2 nm. The OD value obtained was proportional to the concentration of rat PSA.

##### 2.4.1.2. General Chemistry

The general chemistry analysis was performed using the Seamaty microfluidic dry chemistry analyser (Sichuan Province, China). This analyser operation was based on an embedded system and utilised a reagent disc, based on the principle of spectrophotometry to determine the concentrations of 19 parameters in a blood sample. The parameters measured included total protein, globulin, albumin, albumin/globulin ratio, total bilirubin, direct bilirubin, L‐*γ*‐glutamyl transferase (GGT), aspartate aminotransferase (AST), alanine aminotransferase (ALT), alkaline phosphatase (ALP), cholinesterase (CHE), total bile acid (TBA), amylase (AMY), creatine kinase (CK), blood urea nitrogen (BUN), glucose (GLU), total cholesterol (TC), triglycerides (TG), high‐density lipoprotein (HDL), and low‐density lipoprotein (LDL).

##### 2.4.1.3. Haematological Parameter Tests

The Mindray BC 5000 haematology analyser (Shenzhen, China) was used in performing the haematology analyses. The analyser employed the electrical impedance method to determine the count and size distribution of red blood cells (RBC), white blood cells (WBC) and platelets (PLTs). In addition, it used a colorimetric method to determine haemoglobin (HGB).

##### 2.4.1.4. Histopathological Study

Tissues were fixed in appropriate fixatives and processed using Leica TP 1020 tissue processor (Wetzlar, Germany) which employed the routine paraffin embedding procedure. The embedded tissues were subjected to serial sectioning of a thickness of 4 *μ*m using a rotary microtome and were further processed in alcohol‐xylene series, which subsequently got stained with haematoxylin and eosin (H & E). Slides prepared were examined using Olympus CX23 light microscope (Tokyo, Japan).

##### 2.4.1.5. Statistical Analysis

Data was processed in SPSS Version 29. Continuous variables were expressed as mean ± SD. ANOVA was performed to determine areas of statistical differences. Post hoc Bonferroni analysis was then performed to determine specific groups with differences.

##### 2.4.1.6. Ethical Consideration

The protocol was reviewed and approved by the University of Ghana Institutional Animal Care and Use Committee (UG‐ IACUC) with ethics approval number UG‐IACUC 040/23–24. Rats were maintained and treated humanely in line with the five f’s of freedom from hunger and thirst, freedom from discomfort, freedom from pain, injury or disease, freedom from stress and distress, and freedom to express (most) normal behaviour. Additionally, the reduction and refinement principle as part of the 3 R principles used in animal studies were applied.

## 3. Results

From Table 1, ALP and TBA showed significant differences across the groups.

**Table 1:** Effect of *Rauwolfia vomitoria* (*RV*) on liver biochemistry. Low dose 10 mg/kg bwt RV, medium dose (25 mg/kg bwt. *RV*), and high dose (50 bwt kg *RV*).

	**Control**	**Low dose**	**Medium dose**	**High dose**	**p** **value**
ALB	37.8 ± 2.5	34.6 ± 2.8	37.1 ± 1.1	34.2 ± 1.5	NS
TP	74.8 ± 2.5	70.9 ± 4.5	74.1 ± 4.9	71.8 ± 2.8	NS
GLOB	37.0 ± 2.1	36.3 ± 2.6	37.0 ± 4.5	37.5 ± 3.8	NS
A/G	1.0 ± 0.1	1.0 ± 0.1	1.0 ± 0.1	0.9 ± 0.1	NS
TB	1.3 ± 0.9	1.1 ± 1.0	1.6 ± 1.2	2.1 ± 1.3	NS
DB	0.8 ± 0.6	0.7 ± 0.6	0.6 ± 0.4	0.9 ± 0.5	NS
IBIL	0.6 ± 0.4	0.7 ± 0.1	1.0 ± 1.0	1.1 ± 1.2	NS
GGT	3.0 ± 1.3	2.4 ± 0.5	2.4 ± 0.5	3.4 ± 1.1	NS
AST	166.7 ± 34.9	151.2 ± 29.4	189.4 ± 41.5	162.6 ± 80.7	NS
ALT	73.8 ± 35.1	84.4 ± 37.4	61.8 ± 13.1	52.0 ± 18.03	NS
ALP	144.2 ± 29.3^∗^	246.4 ± 66.9^∗^^†^	161.2 ± 48.8^†§^	116.8 ± 16.8^§^	0.008 ^∗^, 0.024^†^, 0.003^§^
TBA	27.9 ± 7.6^∗^^†§^	19.0 ± 5.5^∗^	18.6 ± 4.3^†^	13.2 ± 3.6^§^	0.046 ^∗^, 0.020^†^, 0.004^§^

*Note:* Data expressed as mean ± SD. *n* = 7 per group. Data were analysed by two‐way ANOVA. Similar symbols indicate groups with significant statistical differences based on *p* values. The specific *p* value appears under the *p* value column bearing the same symbol.

Abbreviations: A/G, albumin/globulin ratio; ALB, albumin (g/dL); ALP, alkaline phosphatase (IU); ALT, alanine aminotransferase (IU); AST, aspartate aminotransferase (IU); DB, direct bilirubin (*μ*mol/L); GGT, L‐*γ*‐glutamyl transferase (IU); GLOB, globulin (g/dL); NS, not significant; TB, total bilirubin (*μ*mol/L); TBA, total bile acid (*μ*mol/L); TP, total protein (g/dL).

Post hoc analysis showed differences between the C and the LD, LD and the MD, and MD and the HD. There was a steady decline of ALT from the LD to the HD. Generally, a dose‐dependent decrease was observed.

TBA demonstrated a dose‐dependent decrease. Statistically significant differences were observed between the C and LD and MD and HD. Furthermore, TBA showed significant differences between the C group and the MD group. Finally, there was significance between the C group and the HD group.

From Table 2 which represents the renal function, BUN then creatinine ratio, and uric acid did not show any significant differences. Table 3 represents the lipid profile; TG differences were observed to be significantly low between the C and HD groups (*p* = 0.041). LDL showed an increase dose dependently, with statistical differences emerging between the C and HD groups (*p* = 0.043).

**Table 2:** Effect of *Rauwolfia vomitoria* (*RV*) on renal biochemistry. Low dose (10 mg/kg bwt *RV*) medium dose (25 mg/kg bwt *RV*), and high dose (50 mg/kg bwt kg *RV*).

	**Control**	**Low dose**	**Medium dose**	**High dose**	**p** **value**
BUN	7.1 ± 0.5	6.9 ± 1.1	8.1 ± 1.3	7.5 ± 0.6	NS
Crea	43.9 ± 9.5	41.9 ± 7.0	38.5 ± 13.1	46.0 ± 9.2	NS
BUN/Crea	167.7 ± 37.9	169.9 ± 41.7	242.0 ± 136.4	167.7 ± 29.4	NS
UA	170.8 ± 35.3	194.9 ± 41.8	225.6 ± 67.9	307.9 ± 270.2	NS

*Note:* Values are expressed as mean ± SD. *n* = 7 per group. Data were analysed by two‐way ANOVA.

Abbreviations: BUN, blood urea nitrogen (mmol/L); Crea, creatinine (*μ*mol/L); NS, not significant; UA, uric acid (*μ*mol/L).

**Table 3:** Effect of *Rauwolfia vomitoria* (*RV*) on lipid profile. Low dose (10 mg/kg bwt *RV*), medium dose (25 mg/kg bwt *RV*), and high dose (50 mg/kg bwt *RV*).

	**Control**	**Low dose**	**Medium dose**	**High dose**	**p** **value**
TC	1.8 ± 0.3	1.7 ± 0.4	1.7 ± 0.2	1.8 ± 0.1	NS
TG	1.3 ± 0.3^∗^	1.1 ± 0.5	0.8 ± 0.3	0.7 ± 0.4^∗^	0.041
HDL	1.0 ± 0.1	0.9 ± 0.3	1.0 ± 0.2	1.0 ± 0.115	NS
LDL	0.3 ± 0.1^∗^	0.3 ± 0.2	0.4 ± 0.1	0.5 ± 0.1^∗^	0.043

*Note:* Data expressed as mean ± SD. *n* = 7 per group. Data were analysed by two‐way ANOVA.

Abbreviations: HDL, high‐density lipoprotein cholesterol (mmol/L); LDL, low‐density lipoprotein cholesterol (mmol/L); NS, not significant; TC, total cholesterol (mmol/L); TG, triglycerides (mmol/L).

^∗^Significant difference < 0.05.

Miscellaneous assays were also performed as seen in Table 4. Amylase was significantly elevated between the C and LD groups (*p* = 0.006). Similarly, significant differences were observed between the LD and the MD groups (*p* = 0.006). PSA declined dose dependently but was not statistically significant. Red blood indices (Table 5) did not show significant differences along all the parameters except for haematocrit. Differences in haematocrit showed a decrease between the C and LD groups (*p* = 0.027). On the other hand, there were no statistical differences among the white blood cell indices (Table 6). Organ and relative organ weights did not show differences except for lungs that exhibited a dose‐dependent decrease. Only the absolute organ weights of the lungs were significant (Table 7).

**Table 4:** Effect of *Rauwolfia vomitoria* (*RV*) on other miscellaneous biochemical assays. Low dose (10 mg/kg bwt *RV*), medium dose (25 mg/kg bwt *RV*), and high dose (50 mg/kg bwt *RV*).

	**Control**	**Low dose**	**Medium dose**	**High dose**	**p** **value**
GLU	5.2 ± 2.0^∗^	13.0 ± 6.2^∗^^†§^	4.0 ± 1.3^†^	5.8 ± 1.5^§^	0.001 ^∗^; 0.007^†^; 0.034^§^
AMY	1352 ± 144^∗^^†^	1631 ± 150 ^∗^	1074.2 ± 58.9^†^	1294 ± 282	0.006 ^∗^; 0.006^†^
CK	447.0 ± 222.5	299.8 ± 120.7	486.2 ± 242.6	486.8 ± 290.3	NS
PSA	0.357 ± 0.065	0.437 ± 0.173	0.410 ± 0.126	0.315 ± 0.056	NS

*Note:* Data expressed as mean ± SD. *n* = 7 per group. Data were analysed by two‐way ANOVA. Similar symbols indicate groups with significant statistical differences based on *p* values. The specific *p* value appears under the *p* value column bearing the same symbol.

Abbreviations: AMY, amylase (IU); CK, creatinine kinase (IU); GLU, fasting glucose (mmol/L); NS, not significant; PSA, prostate‐specific antigen (ng/mL).

**Table 5:** Effect of *Rauwolfia vomitoria* (*RV*) on RBC. Low dose (10 mg/kg bwt *RV*), medium dose (25 mg/kg bwt *RV*), and high dose (50 mg/kg bwt *RV*).

**Parameter**	**Control**	**Low dose**	**Medium dose**	**High dose**	**p** **values**
RBC	9.27 ± 0.423	8.60 ± 0.651	9.10 ± 0.441	9.04 ± 0.288	NS
HGB	15.19 ± 0.491	14.00 ± 0.622	15.11 ± 0.674	14.73 ± 0.753	NS
HCT	44.03 ± 1.36^∗^	40.91 ± 1.87^∗^	44.26 ± 2.27	43.13 ± 2.65	0.027
MCV	47.56 ± 1.49	47.74 ± 1.99	48.70 ± 2.62	47.72 ± 2.35	NS
MCH	16.40 ± 0.412	16.34 ± 0.728	16.64 ± 0.800	16.30 ± 0.701	NS
MCHC	34.49 ± 0.453	34.23 ± 0.431	34.17 ± 0.680	34.17 ± 0.450	NS
RDW‐CV	15.67 ± 0.704	15.31 ± 0.773	15.50 ± 0.614	15.23 ± 0.628	NS
RDW‐SD	27.94 ± 1.41	27.36 ± 1.45	28.21 ± 1.16	27.17 ± 0.956	NS
PLT	922 ± 259	857 ± 178	796 ± 170	990 ± 65.9	NS
MPV	7.63 ± 0.411	7.77 ± 0.419	7.69 ± 0.339	7.32 ± 0.248	NS
PDW	14.96 ± 0.127	14.94 ± 0.172	15.06 ± 0.140	14.92 ± 0.160	NS
PCT	0.698 ± 0.180	0.666 ± 0.138	0.612 ± 0.130	0.725 ± 0.048	NS
P‐LCC	110.5 ± 27.51	111 ± 29.38	104.3 ± 24.98	101 ± 17.75	NS
P‐LCR	12.53 ± 3.30	12.97 ± 2.41	13.26 ± 2.50	10.22 ± 1.84	NS

*Note:* Values are expressed as mean ± SD, *n* = 7. Statistically significant at *p* < 0.05. Data were analysed by one‐way ANOVA.

Abbreviations: HCT, haematocrits (%); HGB, haemoglobin concentration (g/dL); MCH, mean corpuscular haemoglobin (pg); MCHC, mean corpuscular haemoglobin concentration (g/dL); MCV, mean corpuscular volume (fL); MPV, mean platelet volume (fL); NS, statistically not significant; PCT, plateletcrit (×10^3^); PDW, platelet distribution width (fL); PLCC, large cell count (fL); P‐LCR, platelet large cell ratio; PLT, platelet count; RBC, red blood cell count (×10^6^); RDW‐CV, red cell distribution width coefficient of variation (%); RDW‐SD, red cell distribution width‐standard deviation (fL).

^∗^Significant difference < 0.05.

**Table 6:** Effect of *Rauwolfia vomitoria* (*RV)* on WBC indices. Low dose (10 mg/kg bwt), medium dose (25 mg/kg bwt), and high dose (50 mg/kg bwt).

**Parameter**	**Control**	**Low dose**	**Medium dose**	**High dose**	**p** **value**
WBC	6.80 ± 2.178	7.31 ± 2.24	5.58 ± 2.85	6.16 ± 3.26	NS
Neu#	1.82 ± 0.648	1.70 ± 0.559	1.62 ± 1.38	1.46 ± 0.444	NS
Lym #	3.79 ± 1.71	4.48 ± 1.65	3.04 ± 1.54	3.74 ± 2.57	NS
Mon#	0.619 ± 0.178	0.689 ± 0.208	0.416 ± 0.223	0.492 ± 0.201	NS
Eos#	0.534 ± 0.231	0.414 ± 0.187	0.484 ± 0.343	0.447 ± 0.232	NS
Bas#	0.030 ± 0.010	0.029 ± 0.028	0.024 ± 0.011	0.023 ± 0.015	NS
Neu%	28.13 ± 8.82	23.41 ± 3.49	27.69 ± 9.92	27.80 ± 10.91	NS
Lym%	53.14 ± 13.92	60.27 ± 7.93	54.73 ± 11.35	55.18 ± 13.58	NS
Mon%	9.61 ± 2.70	9.50 ± 1.06	7.69 ± 2.79	8.67 ± 2.29	NS
Eos%	8.63 ± 4.75	6.36 ± 4.18	9.41 ± 5.88	7.88 ± 2.47	NS
Bas%	0.486 ± 0.212	0.457 ± 0.597	0.486 ± 0.587	0.467 ± 0.250	NS

*Note:* Values are expressed as mean ± SD, *n* = 7. Data were analysed by one‐way ANOVA.

Abbreviations: Bas, basophil count (U/L); Eos, eosinophil (U/L) count; Lym, lymphocytes (%); Mon, monocyte (U/L); Neu, neutrophil count (U/L); NS, statistically not significant; WBC, white blood cell count (×10^3^).

^∗^Statistically significant at *p* < 0.05.

**Table 7:** Organ/relative organ weights of various groups after 90 days of treatment with *R. vomitoria.*

**Organ/rel. organ wt.**	**Control**	**Low dose**	**Medium dose**	**High dose**	**p** **value**
Heart (g)	0.93 ± 0.04	0.86 ± 0.04	0.98 ± 0.06	0.95 ± 0.09	NS
Rel. weight (10^−3^)	3.23 ± 0.18	3.15 ± 0.09	3.91 ± 0.37	3.51 ± 0.28	NS
Liver (g)	8.30 ± 0.31	7.74 ± 0.41	7.66 ± 0.27	7.43 ± 0.49	NS
Rel. weight (10^−3^)	2.89 ± 0.13	2.83 ± 0.09	2.82 ± 0.14	2.75 ± 0.12	NS
Kidney (g)	0.88 ± 0.04	0.84 ± 0.04	0.84 ± 0.05	0.80 ± 0.04	NS
Rel. weight (10^−3^)	3.07 ± 0.17	2.92 ± 0.15	3.09 ± 0.19	2.96 ± 0.12	NS
Spleen (g)	0.53 ± 0.04	0.56 ± 0.02	0.48 ± 0.03	0.58 ± 0.05	NS
Rel. weight (10^−3^)	1.86 ± 0.19	2.05 ± 0.10	1.83 ± 0.21	2.15 ± 0.16	NS
Prostate (g)	0.47 ± 0.04	0.49 ± 0.06	0.57 ± 0.09	0.58 ± 0.07	NS
Rel. weight (10^−3^)	1.64 ± 0.16	1.77 ± 0.19	2.03 ± 0.31	2.16 ± 0.27	NS
Pancreas	0.93 ± 0.07	0.98 ± 0.12	0.67 ± 0.09	0.72 ± 0.06	NS
Rel. weight (10^−3^3)	3.24 ± 0.29	3.76 ± 0.44	2.43 ± 0.34	2.65 ± 0.19	NS
Testes (g)	2.44 ± 0.22	2.68 ± 0.32	2.55 ± 0.15	2.42 ± 0.15	NS
Rel. weight (10^−3^)	8.55 ± 0.93	9.79 ± 1.00	9.49 ± 0.85	8.96 ± 0.47	NS
S. vesicle (g)	1.34 ± 0.14	1.34 ± 0.05	1.09 ± 0.25	1.03 ± 0.17	NS
Rel. weight (10^−3^)	4.64 ± 0.43	4.93 ± 0.15	3.86 ± 0.81	3.88 ± 0.67	NS
Lungs (g)	2.13 ± 0.12^∗^	1.81 ± 0.05^∗^	1.77 ± 0.15	1.62 ± 0.17	0.029 ^∗^
Rel. weight (10^−3^)	7.49 ± 0.64	6.69 ± 0.27	6.53 ± 0.62	5.95 ± 0.51	NS

*Note:* Values are expressed as mean ± SD. *n* = 7 per group.

Abbreviations: NS, statistically not significant; Rel. weight, relative weight.

Figure 1 demonstrates the photomicrographic results of kidney sections. Across the various doses, relatively normal kidney architecture was observed. Figure 2 represents photomicrographs of liver sections. Sections appear to have normal hepatocytes; however, structures around some portal veins in B, C, and D appear altered. Figure 3 represents lung tissues. Photomicrographs reveal distortion of pulmonary architecture at higher doses.

Figure 1Photomicrographs of kidney sections of rats treated with *R. vomitoria* aqueous root back extract for 90 days. (a) Control gp, (b) low dose = 10 mg/kg bwt, (c) medium dose = 25 mg/kg bwt, and (d) high dose = 50 mg/kg bwt. (a, b) Normal architecture with the Bowman’s capsule (arrowed). (c) A defective capsule with loss of Bowman’s space (arrowed). (d) Widening within the capsule (arrowed) (magnification = ×100).

**Figure:**
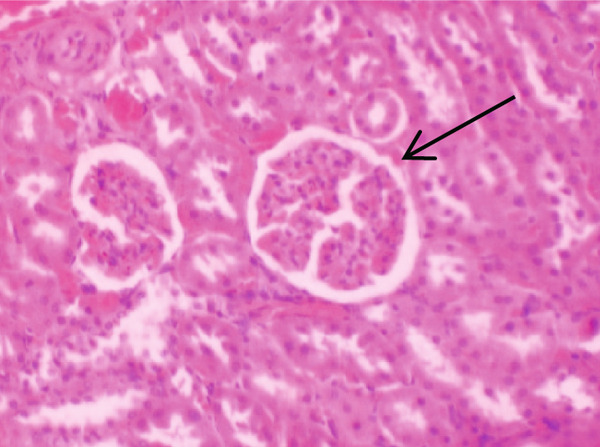
(a)

**Figure:**
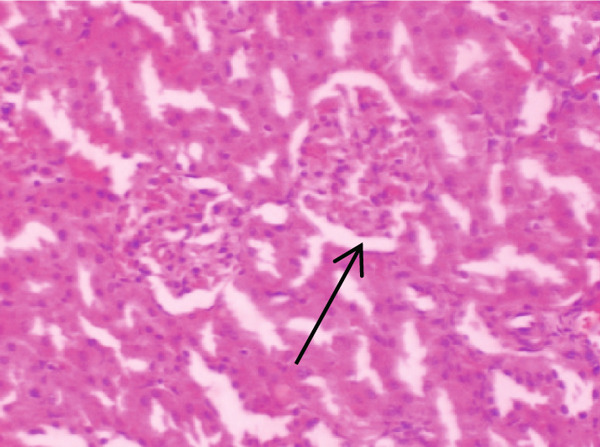
(b)

**Figure:**
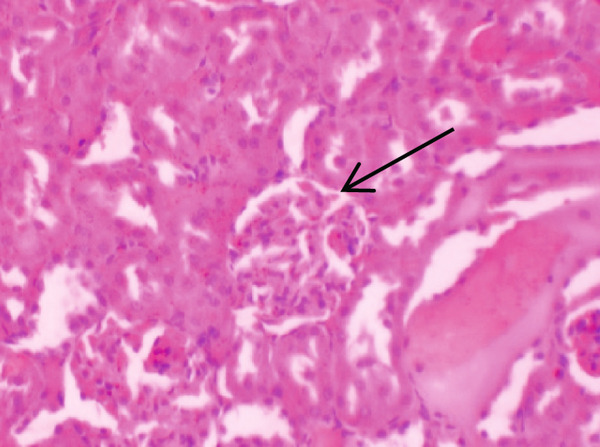
(c)

**Figure:**
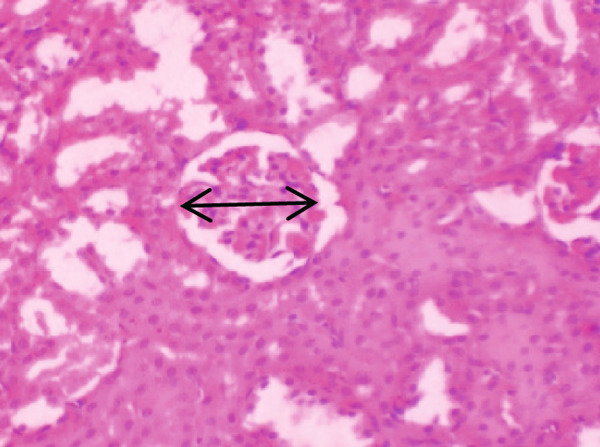
(d)

Figure 2Photomicrographs of liver sections of rats treated with R. vomitoria aqueous root bark extract for 90 days. (a) Control gp, (b) low dose = 10 mg/kg bwt, (c) medium dose = 25 mg/kg bwt, and (d) high dose = 50 mg/kg bwt. All four photomicrographs: (a) a normal‐looking portal triad (arrows); (b) the portal triad (solid arrow): portal veins, red blood cells, and inflammatory cells (broken arrow); (c) more inflammatory cells (solid arrow) within the parenchyma of the liver: the portal vein is infiltrated by red blood cells (broken arrow) and inflammatory cells (arrowheads); and (d) the portal veins looking elongated (circled) and filled with aged red blood cells (arrows) (magnification = ×100).

**Figure:**
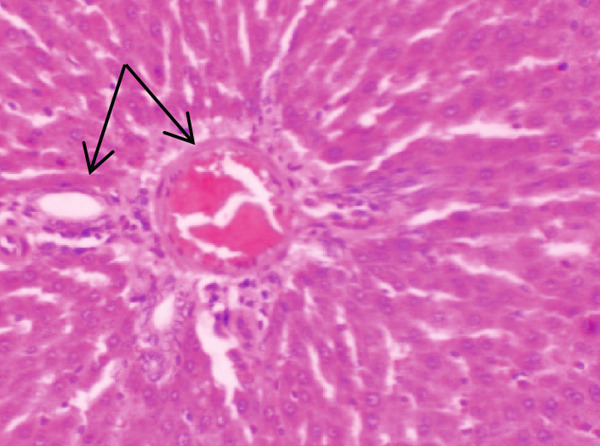
(a)

**Figure:**
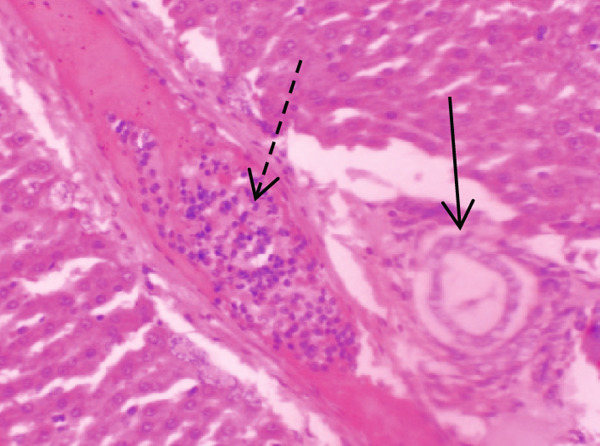
(b)

**Figure:**
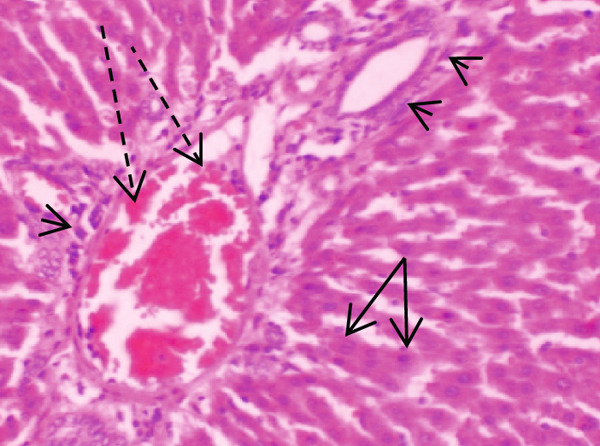
(c)

**Figure:**
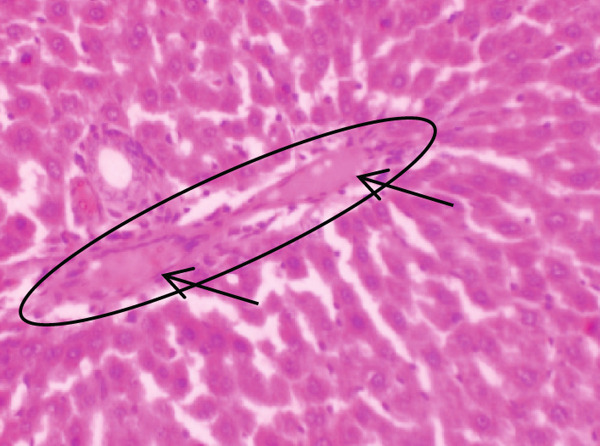
(d)

Figure 3Photomicrographs of lung sections of rats treated with *R. vomitoria* aqueous root back extract for 90 days. (a) Control gp, (b) low dose = 10 mg/kg bwt, (c) medium dose = 25 mg/kg bwt), and (d) high dose = 50 mg/kg bwt. All four photomicrographs show lung parenchyma alveoli, some blood vessels (double arrowheads), and ciliated columnar epithelium of the bronchiole (single arrow). Pulmonary alveoli space is marked by “X.” However, progressive distortion of the alveoli is seen with the treatment groups (photomicrographs b–d) (magnification = ×100).

**Figure:**
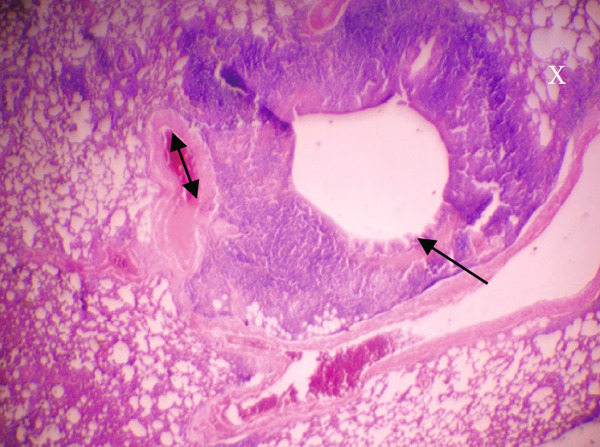
(a)

**Figure:**
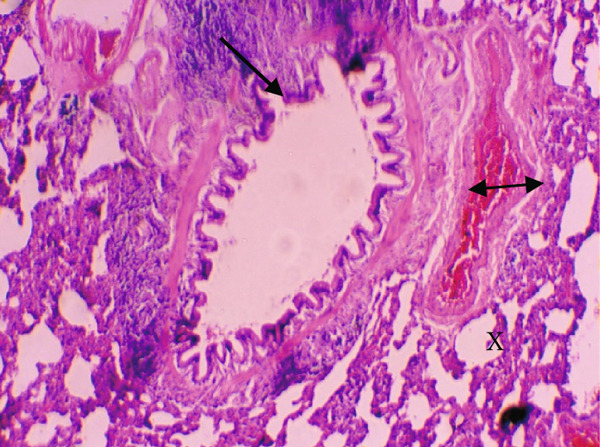
(b)

**Figure:**
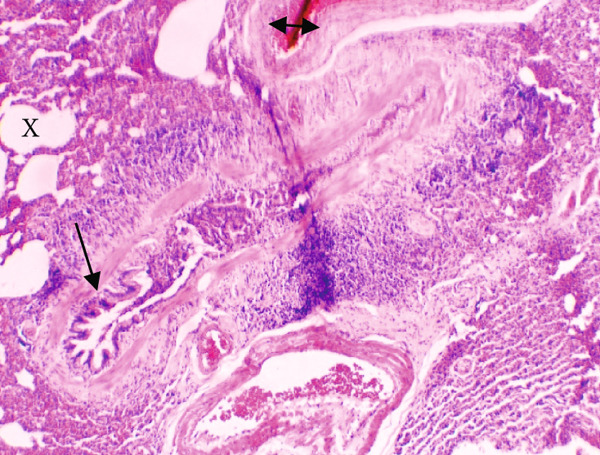
(c)

**Figure:**
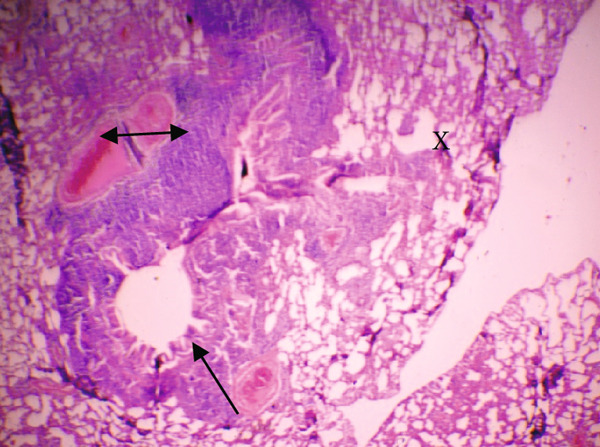
(d)

## 4. Discussion

Allopathic medicines have tremendously improved health and have been well researched. However, other undesirable side effects are sometimes recorded. The quest for natural products for prostamegaly is on the increase. Most of these products have anecdotal evidence of therapeutic efficacy, but few have been well researched for their safety. In the case of natural products for prostate enlargement, *Serenoa repens* [39] and *Croton membranaceus* [40] have been well researched. *R. vomitoria* looks promising with the in vitro and in vivo evidence of reducing 5‐*α*‐reductase, the main enzyme that converts testosterone to dihydrotestosterone, an androgen that fuels prostate enlargement at a therapeutic dose of 20 mg/kg [1]. However, as customary to all toxicity studies, there is the need for a higher and lower dose, aside the therapeutic dose to ascertain its safety.

The point of conjuncture on *R. vomitoria’s* safety hinges on diverse information on its hepatotoxicity. For example, a 7‐day study at 524 mg/kg b.wt. *R. vomitoria* reported of AST and ALT [21]. At doses of 300, 600 and 900 mg/kg bwt, AST and ALT were significantly elevated, but histologically, no lesion was observed [24]. At even higher doses of 1000 and 2000 mg/kg bwt daily, blood and tissue samples did not demonstrate hepatotoxicity [19]. The aqueous leaf extract of *R. vomitoria* showed a significant dose‐dependent increase of AST and ALT at 120 and 300 mg/kg bwt but did not show abnormal histo‐architecture after 21 days of extract administration [41]. On the contrary, others induced hepatotoxicity in experimental rats using carbon tetrachloride (CCl_4_) and applied very LDs (12.3–24.0 mg/kg bwt) of *R. vomitoria*. After 24 h, the authors reported normal liver histology [42].

Looking at the whole profile, from blood to tissue, as well as other related assays of the biliary tree, liver injury is possible. This may have occurred judging from the fact that such biliary tree disorders are evidenced by increased GGT or ALP. Table 3 shows ALP increases in the LD and MD groups that were statistically significant. A reduction of ALP as seen in the high dose group is still suggestive that possibly the high dose escalated the disease process faster; hence, leading to lower protein biosynthesis of which enzymes are of no exception. Indeed, a nondose‐dependent decline in total protein was observed, although not statistically significant. Ultimately, AST and ALT that were not affected could be ascribed to the commencement of the process of liver damage at the cellular level, but not obvious in the blood at the time of sampling.

Although normal histoarchitecture of the liver was observed in another study [41], the current study demonstrates central vein distortion and elongation as well as infiltrations. Histologically, portal veins demonstrated some distortion in this study. Portal tracts infiltrates were also observed (Figure 2). Perhaps these differences observed in that study [41] and the present study could be due to the former using aqueous leaf extract, whilst the latter used the aqueous root back extract.

ALP results were inconsistent compared to total bile acids (TBAs) that decreased dose dependently. Bile acids are synthesised in the liver, and the dose‐dependent decrease is suggestive of possible hepatic dysfunction such as cirrhosis or hepatitis [43]. This resonates with the liver histology that showed features of enlarged portal cells with infiltrations (Figures 2b, 2c, and 2d).

The reduction in the bile acid pool has implications for fat emulsification; hence, the impeded absorption of dietary fat leading to reduced TG synthesis (Table 3). Similar TG reductions were observed in another phytomedicine for BPH such as *Serenoa repens* (Saw palmetto) [44], *Croton membranaceus* [40], and *Urtica dioica* (Nettle Root). TG reduction can also be suggestive of liver dysfunction.

The alteration of the lipid metabolism profile is also seen in a significant dose‐dependent increase of LDL. The poor utilisation of cholesterol to build bile acids implies that cholesterol levels may increase [45]. Lipoprotein particles such as VLDL carry TG. A decrease in TG can result in a decrease in VLDL, which subsequently leads to an increase in IDL and LDL, as seen with some medications or drugs [46]. A dose‐dependent increase in LDL observed in this study may be accounted for by the abovementioned mechanism. Furthermore, there is the possibility of some interference in the LDL receptor activity, leading to low hepatic clearance of LDL and increased circulating LDL.

The aforementioned suggestion agrees with the possibility of reduced digestive enzymes. Table 4 demonstrates that amylase is reduced, especially in the MD and LD groups. Reduced pancreatic function is also related to the poor fat digestion.

For some reason, the LDs of *R. vomitoria* caused a surge in amylase, signifying a possible pancreas dysfunction leading to a possible insulin decline and an elevated glucose level as observed in another study [47]. A reverse trend was seen in the MD and HD groups.

The lungs demonstrated some changes in histoarchitecture as similarly observed by others [23]. However, the kidney did not reveal that much renal dysfunction.

## 5. Conclusion

The study demonstrated a dose‐dependent decrease in lung weights. This is further evidenced by the lung histological abnormalities. The lungs were therefore affected by the extract. With respect to the liver, ALP and TBA increased significantly, demonstrating a liver pathological condition. The liver histology also showed abnormalities, as seen in the photomicrographs. Such presentations of the liver are suggestive of hepatotoxicity. It is obvious that safety concerns can be raised about the use of the aqueous root bark extract of *R. vomitoria* on account of its effect on the lungs and liver. This calls for further investigations.

## Conflicts of Interest

The authors declare no conflicts of interest.

## Author Contributions

P.D.: experimentation and writing; S.A.: conceptualisation and editing; G.A.A.: conceptualisation, writing, and editing; K.B.: supervision; R.A.M.: experimentation and data curation; U.O.: editing and H.R.: histological analysis.

## Funding

No funding was received for this manuscript.

## Data Availability

The data that support the findings of this study are available on request from the corresponding author. The data are not publicly available due to privacy or ethical restrictions.
